# Reliability and validity of the Chinese version of the Gatos Clinical Test questionnaire in patients with severe dementia

**DOI:** 10.3389/fpsyg.2025.1659030

**Published:** 2025-10-20

**Authors:** Yan Zhao, Xuhui Shen, Dandan Mao, Meiying Xu, Shasha Li, Lin Wang, Xueyang Zhao

**Affiliations:** ^1^Department of Nursing, Ningbo Municipal Hospital of Traditional Chinese Medicine (TCM), Affiliated Hospital of Zhejiang Chinese Medical University, Ningbo, China; ^2^Department of Nursing, College of Medical Science, Huzhou University, Huzhou, China; ^3^Huzhou Third People's Hospital, Huzhou, China

**Keywords:** severe dementia, survivability, reliability, validity, factor analysis

## Abstract

**Background:**

The Gatos Clinical Test (GCT) Questionnaire is an effective tool to evaluate the vitality, survival, and maintenance of basic skills potential in patients with severe dementia. However, it has not yet been verified for use in China.

**Objectives:**

This study aims to adapt the GCT questionnaire across cultures and utilize a cross-sectional design to verify its reliability and validity in people with severe dementia in China.

**Methods:**

The original questionnaire was translated into Chinese according to the Brislin two-person translation-return translation method. This study conducted a questionnaire survey on 276 severe dementia patients in China. The patients were 52 to 88 years old, with an average age (78.34 ± 6.42) years old, with 45.3% of men (*n* = 125) and 54.7% of women (*n* = 151). The patients completed the Chinese version of GCT questionnaire and Mini-Mental State Examination and used the item distribution, critical ratio, and correlation coefficient to screen the items. The content validity index was used to evaluate the content validity of the questionnaire. Exploratory factor analysis and confirmatory factor analysis were used to test the construct validity of the questionnaire. The reliability of the questionnaire was evaluated by Cronbach’s alpha coefficient, test–retest reliability and inter-rater reliability evaluation.

**Results:**

The Chinese version of the GCT questionnaire consisted of 14 items. Factor analysis extracted three common factors with a cumulative variance contribution rate of 65.513%. Confirmatory factor analysis demonstrated satisfactory construct validity for the questionnaire. The questionnaire’s content validity index was 0.91. Cronbach’s alpha coefficient was 0.898, test–retest reliability was 0.959, and inter-rater reliability was 0.986.

**Conclusion:**

The Chinese version of the GCT questionnaire has good reliability and validity and can be an effective tool for clinical and community healthcare professionals to assess patients with severe dementia.

## Introduction

Dementia is a group of clinical syndromes that lead to progressive cognitive decline and impaired activities of daily living (Jean and Dotson, 2024). 90% of patients with dementia experience behavioral and psychological symptoms (Aarsland, 2020). World Alzheimer Report (2024) indicated that a new case of dementia emerges every 3 sec globally, with the number of affected individuals projected to reach 139 million worldwide by 2050. Due to the progressive and unpredictable nature of dementia, patients gradually fell into a stupor or coma like a vegetative state, finally succumbing to complications such as pulmonary infections, malnutrition, pressure ulcers, and systemic failure (Moermans et al., 2022; Niznik et al., 2019).

Given the irreversibility of dementia, patients typically require round-the-clock intensive care from caregivers. A study suggests that long-term care for aggressive dementia patients can lead to caregiver fatigue, fear, and exhaustion (Sheikh et al., 2022). Progressive speech impairments and cognitive deficits significantly hinder patient-caregiver communication, caregivers and healthcare professionals find it challenging to accurately identify patients’ care needs. This can result in withdrawal from social interactions, depression, social isolation for the person with dementia, and increased caregiver burden (Dooley et al., 2025). Frustration intensifies when exhaustive caregiving efforts prove ineffective in alleviating disease progression, potentially triggering abusive behaviors (e.g., verbal assaults or neglect) and abandonment ideation toward patients. Such maladaptive responses inflict multidimensional harm, encompassing physical/psychological trauma and erosion of human dignity in patients (Steinsheim et al., 2022; Browning et al., 2024).

Notably, caregivers’ excessive negative emotions and neglect of patients’ needs and residual survival capacities are significantly associated with accelerated disease progression. As the disease advances, traditional cognitive assessment tools such as Mini-Mental State Examination (MMSE) and Montreal Cognitive Assessment (MoCA) exhibit “floor effects,” failing to accurately characterize the features and evolutionary patterns of functional impairment in severe dementia. This superficial assessment framework substantially restricts the potential for meaningful caregiver-patient communication. Current tools for evaluating severe dementia-including Severe Impairment Battery (SIB), Severe Impairment Battery Short Version (SIB-S), Severe Mini-Mental State Examination (SMMSE), Test for Severe Impairment (TSI), and Severe Cognitive Impairment Profile (SCIP)— primarily focus on cognitive domains (Saxton, 1990; Saxton et al., 2005; Harrell et al., 2000; Albert and Cohen, 1992; Peavy et al., 1996). These instruments predominantly target populations with MMSE scores of 0–9, often requiring complex multi-item administration. Operational challenges persist, with certain tools demonstrating educational bias and limited sensitivity in tracking longitudinal cognitive changes.

In reality, despite a significant cognitive decline in patients with advanced dementia, certain functional capacities such as nonverbal communication, short-term memory retention, color discrimination, and nociceptive perception may remain partially preserved to varying degrees. However, it is crucial to note that the execution of even simple arithmetic tasks can be heavily impaired in severe dementia, as demonstrated in neuropsychological studies (Giannouli and Tsolaki, 2023a; Giannouli, 2024; Giannouli and Tsolaki, 2023b).

Therefore, to precisely identify disease progression, residual survival capacities, and preserved functional potential in patients with severe dementia–while reducing caregivers’ neglect/abusive behaviors and ensuring end-stage quality of life and dignity—there is a critical need to implement validated multidimensional assessment tools. Although the Gatos Clinical Test (GCT) Questionnaire shows promise, its psychometric properties remain unverified in Chinese populations. Therefore, this study translated and culturally adapted the GCT questionnaire into Chinese for comprehensive psychometric evaluation.

## Research purpose

Patients with severe dementia receive relatively low attention in China, primarily due to their low sensitivity to commonly used assessment tools, and the lack of understanding regarding this group often leads to their neglect. The GCT questionnaire has not been validated in China, which to some extent restricts the country’s ability to comprehensively address the progression of elderly dementia patients’ conditions and changes in cognitive function. This study aims to translate, back-translate, and culturally adapt the English version of the GCT questionnaire, revising the Chinese version based on the medical cultural context of our country, and evaluating the reliability and validity of the Chinese version of the GCT questionnaire. It seeks to explore its applicability in the population of severe dementia patients in our country through objective quantitative indicators, track changes in the condition of severe dementia patients, assess their potential for survival and maintenance of basic skills, and guide for maintaining the quality of life of patients with severe dementia and implementing nursing measures.

## Methods

### Design and participants

This prospective cross-sectional study employed convenience sampling to enroll 276 patients with severe dementia from Huzhou Third People’s Hospital. Inclusion criteria were: (a) Diagnosis of dementia confirmed by psychiatrists through neuropsychological assessments and medical history review, by ICD-10 criteria; (b) MMSE scores of 0–2; (c) Signed informed consent from legal guardians. Exclusion criteria (meeting any single item): (a) Severe auditory/visual impairment; (b) Mobility limitations due to fractures or other somatic conditions; (c) Comorbid severe psychiatric disorders; (d) Neurological diseases causing impaired facial expressivity.

We collected sociodemographic data from all participants, who completed assessments using both the Mini-Mental State Examination (MMSE) and the Chinese version of the Gatos Clinical Test Questionnaire (GCT). To evaluate inter-rater reliability, two researchers independently and simultaneously administered the GCT to patients item-by-item without verbal communication during the process; all questionnaires were retrieved immediately post-assessment. For test–retest reliability analysis, a randomly selected subgroup of 12 patients underwent repeat GCT evaluations using the Chinese version after a one-week interval.

### Sample size calculation

The sample size for this validation study was estimated based on the subject-to-item ratio recommended for factor analysis. According to the rule of thumb for sample size, the scale requires a minimum of 10 subjects per item for factor analysis (Myers et al., 2011). In this study, there were 14 items, and considering a further 10% null error, the estimated sample size was 154 cases. In addition, the sample size for the reference exploratory factor analysis should be at least ≥ 100 cases, and the sample size for the confirmatory factor analysis should be ≥ 200 cases. To ensure a robust factor structure and achieve stable parameter estimates, a total of 276 questionnaires were distributed, and 276 questionnaires were validly returned and included in the analysis, with a valid return rate of 100%. Of these, 126 questionnaires were randomly selected for exploratory factor analysis (EFA), and the remaining 150 were selected for confirmatory factor analysis (CFA).

### Recruitment and testing procedure

Participants were consecutively recruited from the inpatient geriatric psychiatry units of Huzhou Third People’s Hospital between [January, 2019] and [May, 2020]. The attending physicians initially identified potential participants who met the clinical diagnostic criteria for severe dementia. Their legal guardians were then approached by the research team, provided with detailed information about the study’s aims and procedures, and invited to participate.

All assessments were conducted at the bedside in a quiet and well-lit room, with minimal disruptions. The MMSE was administered first, followed by the GCT questionnaire. Each assessment session lasted approximately 20–30 min. To prevent fatigue, breaks were offered if needed. The researchers administering the tests were trained research nurses who were proficient in the use of both instruments. To ensure consistency, a standardized script was used to introduce and explain each task to the participants and their caregivers.

## Instruments

### General information questionnaire

A patient sociodemographic characteristics questionnaire was developed through a comprehensive process involving literature review and expert panel discussions. This instrument captures essential demographic variables including but not limited to: name, gender, age, occupation, clinical diagnosis, educational level, marital status, and residential region.

### Mini-Mental Status Examination (MMSE)

The MMSE is internationally recognized as the gold-standard global cognitive screening instrument, comprising assessments across six cognitive domains (Steinsheim et al., 2022). The total MMSE score ranges from 0 to 30, with scores ≤ 27 indicating cognitive impairment. Specifically, scores of 21–26 classified as mild dementia, 10–20 as moderate dementia, and ≤ 9 as severe dementia.

### Gatos Clinical Test (GCT) questionnaire

The GCT questionnaire, jointly developed by the University of Athens, the University of Thessaly, and the Neurological clinic “Agios Georgios,” is designed to comprehensively assess patients with severe dementia (MMSE score ≤ 2) (Tsoucalas et al., 2015). The GCT questionnaire comprises two components: 6 general information items capturing demographic and pathological characteristics (A. Hearing, B. Eyesight, C. General status, D. Patient behavior, E. Facial expressions, F. Walking-movement), and 14 domain-specific items systematically evaluating cognitive function, daily living abilities, and somatic manifestations. The total score of GCT questionnaire ranges from 0 to 26 points, with higher scores indicating better clinical status greater survival capacity, and preserved functional potential. Scoring thresholds are stratified as follows: 0–9.5 points (poor status), 9.5–18.5 points (moderate status), and 18.5–26 points (excellent status). Psychometric analyses confirm its robust reliability and validity. Notably, the questionnaire demonstrates high specificity and sensitivity, effectively distinguishing patients with MMSE scores of 0 from those scoring 1–2. This instrument shows strong potential as a validated tool for assessing survival capacity and preserved functional competencies in advanced dementia populations.

## Procedures

### Translation and culture adaptation of the questionnaire

Following authorization from the original author, Dr. Gregory Tsoucalas, the GCT questionnaire underwent rigorous translation and cultural adaptation using the Brislin model translation-back translation methodology (Brislin, 1976). First, two bilingual translators Master of Nursing Science and a Master of English Linguistics-independently translated the original English version into Chinese. An overseas-trained clinical nursing expert then reconciled discrepancies between the two translations through iterative revisions to produce a preliminary Chinese version ensuring semantic equivalence and conceptual fidelity. Subsequently, two additional bilingual translators, unfamiliar with the original instrument but specialized in dementia research, independently back-translated the Chinese draft into English. A nursing scholar with international training compared these back-translated versions against the original questionnaire, resolving ambiguities to generate a consolidated English draft. Six native English-speaking international students then evaluated conceptual consistency between the back-translated and original questionnaires using a 4-point Likert scale (1 = inconsistent, 4 = fully consistent), achieving a back-translation consistency rate of 92.8%. Finally, the back-translated draft was submitted to Dr. Tsoucalas for validation of conceptual alignment, semantic accuracy, and cultural appropriateness. Any discrepancies identified underwent cyclic re-translation and re-back-translation until full concordance with the original instrument was attained.

To ensure cultural appropriateness within the Chinese context, ten experts were recruited to evaluate the questionnaire’s cultural adaptation and content validity, with revisions made to linguistically challenging items. Expert selection criteria included: (1) extensive professional expertise and high academic qualifications; (2) ≥ 10 years of clinical experience in geriatric psychiatry; (3) possession of a bachelor’s degree or higher combined with mid-level or above professional certification.

### Statistical analysis

Statistical analyses were performed using SPSS 23.0 and AMOS 24.0 with statistical significance set at *p* values < 0.05. Descriptive statistics were applied to socio-demographics data, while item screening employed frequency analysis, independent samples *t*-tests, and Pearson correlation coefficients. Content validity was evaluated using the content validity index (CVI). Construct validity was assessed through Pearson correlation analysis, exploratory factor analysis (EFA) and confirmatory factor analysis (CFA). Reliability was determined via Cronbach’s alpha coefficient, test–retest reliability, and inter-rater reliability.

### Item analysis

#### Item distribution

Examine the distribution trend of each item option and delete any item with a selection rate of more than 80% (Yang et al., 2022).

#### Critical ratio

Following the calculation of total questionnaire scores, participants were ranked in descending order and divided into high-score (top 27%) and low-score (last 27%) groups. Independent samples *t*-tests were conducted to compare item-level score differences between these groups. Items demonstrating a CR value below 3.0 were flagged for potential elimination during psychometric refinement (Zhang et al., 2024).

#### Correlation coefficient

The validity and independence of questionnaire items were evaluated by calculating Pearson correlation coefficients between each item and the total score, as well as among individual items. Homogeneity and discriminant validity were considered satisfactory when item-total correlations exceeded 0.4 and inter-item correlations remained below 0.8 (Besmens et al., 2025; Yeom and Lee, 2024).

### Validity analysis

#### Content validity

Ten experts specializing in geriatric psychiatry were invited to evaluate the relevance of each questionnaire item to the measurement objectives. A 4-point Likert scale was employed for item-level assessment, with scores defined as: 1 = “irrelevant,” 2 = “weakly relevant,” 3 = “moderately relevant,” and 4 = “highly relevant.” Content validity was derived from expert ratings. The item-level content validity index (I-CVI) was calculated as the number of experts assigning scores of 3 or 4 divided by the total number of participating experts. The scale-level content validity index (S-CVI) was calculated as the mean of all I-CVI scores. According to established standards, an I-CVI of ≥ 0.78 and an S-CVI of ≥ 0.90 are considered indicative of good content validity for the scale (Polit and Beck, 2006).

#### Construct validity

Construct validity refers to the accuracy and effectiveness of an assessment tool in measuring a specific object or variable (Long and Huang, 2025). In this study, construct validity was assessed using exploratory factor analysis and confirmatory factor analysis. The 276 participants were randomly divided into two groups for different analyses: 126 individuals for EFA, and the another 150 for CFA. Before factor analysis, the Kaiser-Meyer-Olkin (KMO) test and Bartlett’s sphericity test were performed to evaluate the suitability of the data for factor analysis (Lacobucci et al., 2022). A KMO value > 0.6 and Bartlett’s sphericity test < 0.05 indicated that the data were suitable for factor analysis (Sun et al., 2025). Principal component analysis (PCA) was used to extract common factors, retaining those with a cumulative variance contribution rate exceeding 50% (Shi et al., 2022). During EFA, maximum variance rotation was applied, and items with factor loadings < 0.4 were removed (Zhang et al., 2024). CFA was employed to assess the consistency between the model structure and the exploratory factor structure, and the following indices were used to evaluate the model’s fitness: (1) χ^2^/df; (2) RMSEA; (3) RMR; (4) CFI; (5) NFI; (6) TLI. A model with χ^2^/df less than 3, RMSEA less than 0.08, RMR less than 0.05, and a CFI, NFI and TLI more than 0.90 is considered acceptable (Sun et al., 2025).

### Reliability analysis

#### Internal consistency reliability

To examine the inter-item correlations and homogeneity of the questionnaire, Cronbach’s alpha coefficient was employed to assess the internal consistency among items. The instrument demonstrated acceptable reliability when the overall Cronbach’s alpha coefficient exceeded 0.7, with higher values indicating stronger internal consistency (Gondim et al., 2025).

#### Test–retest reliability

One week later, 12 patients were randomly selected to undergo repeated testing to evaluate the test–retest reliability of the questionnaire. The test–retest reliability coefficient, calculated as the Pearson correlation coefficient between the two measurements, demonstrated satisfactory stability with a value exceeding 0.7 (He et al., 2024).

#### Inter-rater reliability

Inter-rater reliability was assessed using the Pearson correlation coefficient to quantify agreement between independent evaluators. The questionnaire coefficient exceeding 0.7 indicates minimal variability in assessment outcomes across raters (Zhao et al., 2024).

## Results

### Socio-demographic characteristics

A total of 276 patients were formally surveyed, with all questionnaires collected and a 100% valid response rate. The cohort comprised 155 cases (56.2%) of Alzheimer’s disease (AD), 77 cases (27.9%) of vascular dementia (VD), 33 cases (12.0%) of mixed dementia (MD), and 11 cases (4.0%) of other dementia subtypes. The sample included 125 males (45.3%) and 151 females (54.7%), with ages ranging from 52 to 88 years and a mean age of 78.34 ± 6.42 years. Detailed socio-demographic characteristics are presented in Table 1.

**Table 1:** Demographic characteristics of participants (*N* = 276).

Variable	N (%)	%
Gender		
Male	125	45.3
Female	151	54.7
Age group (years)		
50–65	16	5.8
66–70	23	8.3
71–80	85	30.8
>80	152	55.1
Occupation		
Unemployed	29	10.5
Mental workers	83	30.1
Manual laborers	164	59.4
Type of disease		
AD	155	56.2
VD	77	27.9
MD	33	12.0
Other	11	4.0
Educational		
Illiterate	52	18.8
Primary	81	29.3
Junior High	95	34.4
Higher	48	17.4
Marital status		
Single	8	2.9
Married	178	64.5
Divorced	21	7.6
Widowed	69	25.0
Residential address		
Urban	154	55.8
Rural	55	19.9
Suburbs	67	24.3

### Item analysis

Frequency analysis was performed on the 14 items of the Chinese version of the GCT questionnaire, revealing that the selection rate of any single option per item remained below 80%. Total scores were calculated and ranked, with the top 27% classified as the high-score group and the bottom 27% as the low-score group. Independent samples *t*-tests comparing item scores between these groups demonstrated CR ranging from 6.319 to 37.331 (*p* < 0.01), all exceeding the threshold of 3.0, thus retaining all items. Item-total correlations ranged from 0.487 to 0.895 (all > 0.4), indicating strong homogeneity. Inter-item correlations varied between 0.095 and 0.841, with only Items 5 and 13 showing a relatively high correlation coefficient of 0.841; all other inter-item correlations remained below 0.8, confirming adequate independence.

### Validity analysis

#### Content validity

The content validity of the Chinese version of the GCT questionnaire was evaluated by 10 experts using the CVI. Results indicated that five items (Items 2, 3, 6, 7, and 8) received unanimous ratings of 3 or 4 (“moderately relevant” or “highly relevant”) from all experts. Furthermore, 10 items achieved I-CVI scores ≥ 0.90 (i.e., rated 3 or 4 by ≥ 9 experts). The I-CVI for the Chinese GCT questionnaire ranged from 0.80 to 1.00, with an S-CVI of 0.91, indicating that the questionnaire’s content validity was good.

#### Construct validity

##### Exploratory factor analysis

**Figure 1:**
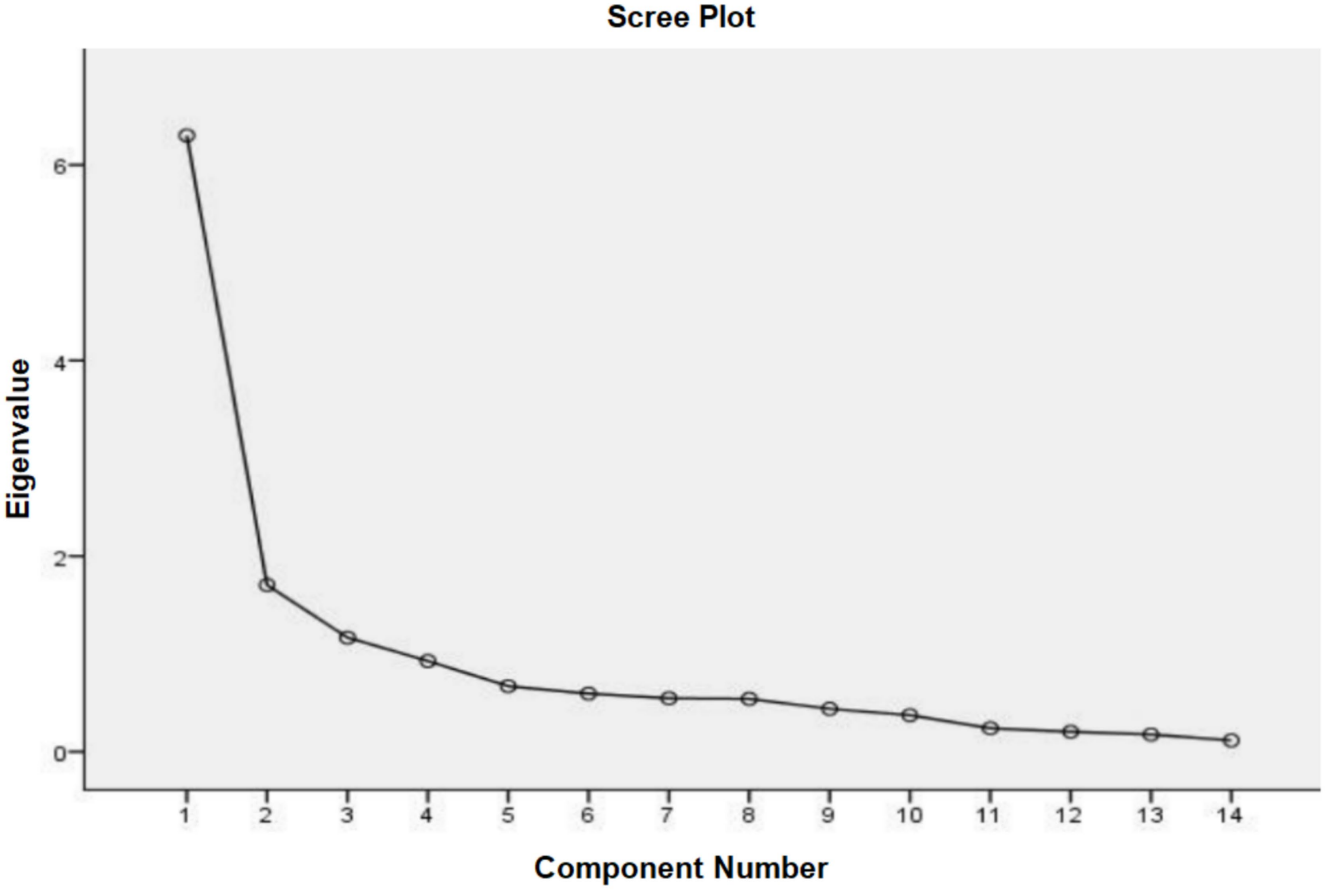
Scree plot of the GCT.

The results showed that KMO = 0.877 (> 0.60), Bartlett’s spherical test χ^2^ = 1004.191 (*p* < 0.001), and df = 91, confirming the suitability of the questionnaire for EFA. PCA with varimax orthogonal rotation was performed without restricting the number of factors. Combined with scree plot analysis (Figure 1), three common factors with eigenvalues exceeding 1 were ultimately extracted, demonstrating factor loadings ranging from 0.519 to 0.902 and a cumulative variance contribution rate of 65.513%. Based on the latent characteristics of these factors and the dimensional structure of the original English questionnaire, the three factors were named as follows: “Autonomy/Alertness” (Items 1, 2, 3, 4, and 6; 5 items), “Intuition/Cognition” (Items 7, 8, 9, 10, and 11; 5 items), and “Somatokinetic Function//Sleep” (Items 5, 12, 13, and 14; 4 items). The rotated component matrix is presented in Table 2.

**Table 2:** Factor loadings after rotated of each item (*N* = 126).

Item	Factor 1	Factor 2	Factor 3
Item_6	**0.864**		
Item_4	**0.818**		
Item_2	**0.768**		
Item_1	**0.736**		
Item_3	**0.688**		
Item_9		**0.809**	
Item_7		**0.665**	
Item_10		**0.640**	
Item_8		**0.615**	
Item_11		**0.519**	
Item_13			**0.902**
Item_5			**0.871**
Item_12			**0.667**
Item_14			**0.654**
Eigenvalues	6.300	1.704	1.167
% Variance	45.002	57.176	65.513

The bolded values represent the factor loading coefficients for each item after orthogonal rotation. Factor loadings ≥ 0.4 were considered acceptable.

##### Confirmatory factor analysis

**Figure 2:**
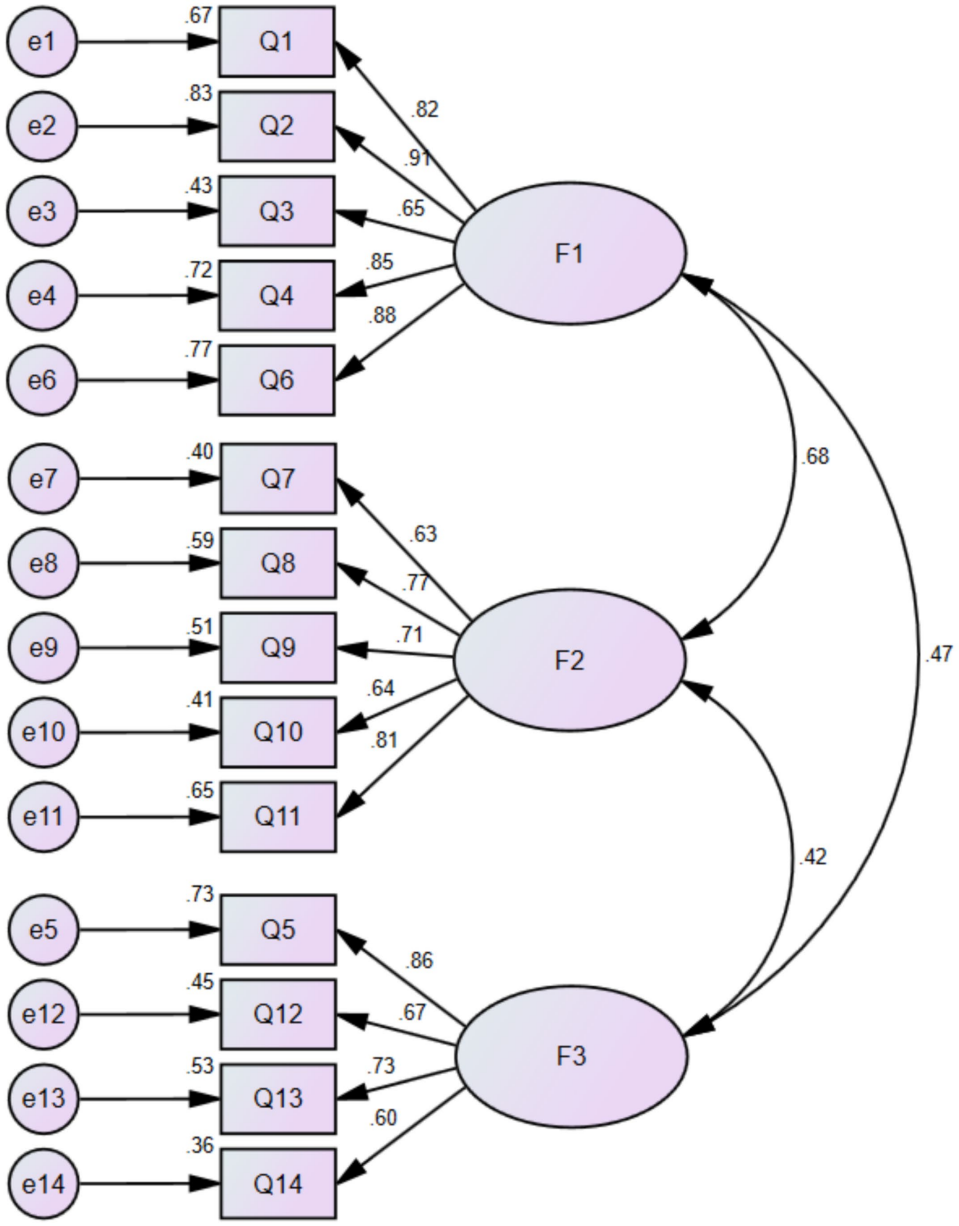
CFA factor loadings of the Chinese version of GCT questionnaire.

The model fit parameter values of χ^2^/df = 1.508, RMSEA = 0.058, RMR = 0.027, CFI = 0.966, NFI = 0.907 and TLI = 0.958. The factor loading values ranged from 0.60 to 0.91, as shown in Figure 2.

##### Reliability analysis

The Chinese version of the GCT questionnaire demonstrated strong internal consistency, with an overall Cronbach’s alpha coefficient of 0.898. Subscale reliability coefficients were 0.901 (Autonomy/Alertness), 0.734 (Intuition/Cognition), and 0.850 (Somatokinetic Function//Sleep). Test–retest reliability was evaluated in 12 patients with severe dementia after a one-week interval, yielding an overall test–retest reliability coefficient of 0.959, with subscale coefficients ranging from 0.882 to 0.953. Inter-rater reliability was assessed by two independent researchers evaluating 30 patients simultaneously, resulting in an overall inter-rater correlation coefficient of 0.986. Subscale inter-rater coefficients ranged from 0.877 to 0.976, confirming excellent inter-rater stability across all dimensions of the Chinese GCT questionnaire.

## Discussion

Dementia is an irreversible neurodegenerative disorder characterized by progressive deterioration of cognitive function. Traditional assessment tools exhibit limited capacity for longitudinally tracking cognitive decline in severe dementia, failing to monitor disease progression and therapeutic efficacy, which perpetuates clinical neglect by healthcare professionals and caregivers–a detrimental cycle. The GCT questionnaire, developed by Dr. Gregory Tsoucalas and colleagues through over three decades of in-depth analysis involving 15,000 + dementia cases, was formally validated in 2015 to evaluate disease trajectory, survival capacity, and preserved functional potential in severe dementia (MMSE scores 0–2) across 500 patients (Tsoucalas et al., 2015). Our cross-cultural adaptation demonstrates that the Chinese GCT questionnaire exhibits robust discriminative power and homogeneity, with excellent reliability and validity. This instrument enables clinicians to systematically assess disease progression, identify residual cognitive functions, and evaluate preserved survival-related competencies in severe dementia, thereby informing personalized therapeutic and caregiving strategies.

Item analysis, a critical process for refining questionnaires by eliminating redundant items and optimizing quality, revealed favorable psychometric properties in the Chinese GCT questionnaire. All 14 items demonstrated adequate discriminative power, with selection rates for any single response option below 80% and no significant skewness in item distribution. CR between high-score and low-score groups exceeded 3.0 across all items (range: 6.32–37.33, *p* < 0.01), confirming robust discriminative capacity. Strong item-total correlations (0.487–0.895, all > 0.4) indicated excellent construct homogeneity. Inter-item correlations ranged from 0.095 to 0.841, with only Items 5 and 13 showing a relatively high correlation coefficient (0.841), suggesting potential redundancy due to overlapping constructs. Following established psychometric protocols, items with ≥ 1 suboptimal metric were flagged for elimination. However, through an expert panel review emphasizing clinical utility and the instrument’s comprehensiveness, Items 5 and 13 were retained despite their elevated correlation, pending further validation in expanded samples. Consequently, all 14 items were preserved in the final Chinese GCT questionnaire.

Validity is about what an instrument measures and how well it does so (Ahmed and Ishtiaq, 2021). This study primarily evaluated the content validity and construct validity of the Chinese version of the GCT questionnaire. With 10 experts participating, the I-CVIs ranged from 0.80 to 1.00 (all > 0.78), and the S-CVI was 0.91, confirming a strong consensus that the items adequately reflect the target constructs. Construct validity, reflecting the intrinsic properties of the measurement tool, was assessed through EFA and CFA. Following established criteria (cumulative variance > 50%, factor loadings > 0.4, and correlations > 0.4), three common factors were extracted, accounting for 65.513% of the cumulative variance. Factor 1 (“Autonomy/Alertness”) included Items 1, 2, 3, 4, and 6 (loadings: 0.688–0.864), Factor 2 (“Intuition/Cognition”) comprised Items 7, 8, 9, 10, and 11 (loadings: 0.519–0.809), and Factor 3 (“Somatokinetic Function//Sleep”) encompassed Items 5, 12, 13, and 14 (loadings: 0.654–0.902). While the dimensional structure of the Chinese GCT questionnaire fully aligns with the original questionnaire, minor adjustments were made to item-factor assignments to enhance scientific rigor: Item 5 was reallocated to Factor 3 due to its stronger relevance to “Somatokinetic Function//Sleep,” and Item 6 was assigned to Factor 1 to better reflect “Autonomy/Alertness,” demonstrating improved construct alignment and robust construct validity compared to the original instrument. CFA results indicated a sound fit for the three-factor model, with all fit indices meeting acceptable thresholds. This model effectively explained the relationships among the variables, thus demonstrating the structural integrity of the scale.

Reliability refers to finding the same result over time, reflecting the true representation of the measured characteristics (Olmsted, 2024). This study evaluated internal reliability through internal consistency analysis and external reliability via test–retest and inter-rater reliability. The Chinese GCT questionnaire demonstrated strong internal consistency, with a total Cronbach’s alpha of 0.898 (exceeding the original questionnaire’s 0.84) and subscale coefficients of 0.734–0.901 (all > 0.7), indicating a higher level of internal consistency in the Chinese GCT questionnaire. Test–retest reliability, a key metric for temporal stability, yielded an overall coefficient of 0.959, with subscale coefficients of 0.951, 0.953, and 0.882, confirming robust stability over time. Inter-rater reliability analysis revealed an overall coefficient of 0.986 and subscale coefficients of 0.877–0.976 (all > 0.7), indicating high inter-rater agreement. Across all metrics–internal consistency, test–retest reliability, and inter-rater reliability—the Chinese GCT questionnaire outperformed the original instrument, demonstrating superior internal and external stability for clinical application.

The findings revealed that Item 1 (“Method of ingesting food”) received notably high scores, indicating relatively preserved swallowing function in patients with severe dementia. Caregivers should prioritize monitoring swallowing capacity to avoid force-feeding practices, thereby reducing risks of choking, aspiration, and subsequent pulmonary infections. Patients demonstrated the ability to respond to simple questions and single-step commands but exhibited limited responsiveness to sequential instructions, suggesting that healthcare providers and caregivers should adopt phased, step-by-step guidance during interactions rather than using complex or lengthy statements. Notably, the study identified preserved facial recognition of family members among patients, potentially linked to retained emotional attachment, which provides compelling evidence to bolster caregiver confidence and encourage active familial involvement in therapeutic and caregiving processes.

Patients with severe dementia, due to their profound dependency, are highly vulnerable to elder maltreatment, including neglect, psychological, or physical abuse (Giannouli, 2022). This study demonstrates that regardless of the MMSE score (0–2), these patients retain awareness and nociception, and remain capable of experiencing distress despite their inability to verbalize discomfort. Key risk factors for maltreatment include caregiver exhaustion, frustration, and insufficient training or support. The patients’ inability to express needs or discomfort often leads to unintentional neglect, such as unaddressed pain, poor hygiene, or inadequate nutrition. The GCT questionnaire objectively identifies preserved abilities, such as pain perception, and serves as an instrumental tool for healthcare professionals to educate caregivers—enabling them to recognize signs of physical discomfort through facial cues. Such education enhances caregivers’ empathy, corrects misconceptions regarding patients’ level of awareness, and emphasizes the importance of treating patients as sentient and dignified individuals, thereby contributing to abuse prevention.

Beyond cognitive impairment, patients with severe dementia bear a significant psychosocial burden, which stems from pervasive social stigma and cultural misconceptions (Giannouli, 2017; Makri and Giannouli, 2022). These individuals are often socially isolated and perceived as “empty shells,” leading to a loss of personal integrity and dignity. This stigmatization also extends to family caregivers, evoking feelings of shame, social withdrawal, and reluctance to seek external support (Noguchi et al., 2025). A lack of awareness and cultural taboos further contribute to the perception of dementia as an inevitable and shameful part of aging, rather than as a neuropathological condition. Such misconceptions hinder access to necessary support and services for both patients and their families. Our findings challenge this narrative by demonstrating that patients with severe dementia retain certain functional and conscious capacities. Shifting the conceptualization of severe dementia from “total loss” to “preserved abilities” can help mitigate prejudice, facilitate social inclusion, and ultimately improve the quality of life for patients and their caregivers.

The validation of the GCT questionnaire in the Chinese population underscores the importance of a cross-cultural perspective in the care of severe dementia. Perceptions of aging, dementia, filial piety, and definitions of “abuse” or “neglect” are deeply embedded in cultural contexts (Makri and Giannouli, 2022). In China, filial piety—a core cultural value—helps prevent elder abandonment, yet it also places considerable pressure on the single-child generation, potentially increasing caregiver burden and the risk of maltreatment. Moreover, cultural norms shape the identification of problematic behaviors: actions considered inappropriate in one cultural setting may be tolerated in another. By providing an objective and culturally adapted assessment framework, the GCT tool can, to some extent, transcend subjective cultural variations and enable standardized evaluation of patient needs and caregiver burden across different cultural backgrounds.

Based on the study findings, we propose several pathways to prevent abuse. First, the GCT questionnaire should be integrated into routine clinical practice to establish a “capability profile” for each patient with severe dementia. This profile can inform the development of individualized care plans centered on preserved abilities, thereby making care more manageable and enhancing caregivers’ sense of accomplishment. Second, assessment results should be incorporated into family psychoeducation programs to correct misperceptions and reduce stigma (Giannouli and Tsolaki, 2022). Finally, high caregiver burden represents a key risk factor for abusive behavior. Therefore, it is essential to help families access external care resources and establish professional support systems to mitigate this risk at its source.

Future research should explore the direct relationship between specific functional impairments identified by the GCT questionnaire—such as impaired pain expression—and incidents of neglect. Furthermore, longitudinal studies are needed to track how the progression of functional decline influences caregivers’ psychological well-being and the risk of abuse. Such studies are crucial for developing targeted, evidence—based interventions to safeguard the rights, quality of life, and dignity of this vulnerable population.

### Limitations

This study has several limitations. The sample selection exhibited geographical constraints due to time and resource limitations, potentially affecting population representativeness. The absence of criterion-related validity testing reflects the current lack of widely adopted severe dementia assessment tools in China. Future multi-center studies with larger, more diverse samples should: (1) investigate determinants influencing survival capacity and preserved functional potential in severe dementia; (2) establish evidence-based interventions tailored to this population. Such efforts would address existing methodological gaps while enhancing the clinical applicability of dementia assessment tools.

## Conclusion

The Chinese version of the GCT questionnaire comprises six general parameters related to patients’ pathological characteristics and 14 domain-specific items. Validation studies confirmed its robust reliability and validity, systematic assessment coverage, and time-efficient administration. The instrument requires minimal linguistic demand from patients and is readily applicable at the bedside or in outpatient settings, demonstrating broad utility for evaluating and staging severe dementia. The questionnaire features a well-defined scoring system that enables healthcare professionals and caregivers to accurately assess the vitality, survival, and potential for maintaining basic skills in patients with severe dementia. It facilitates the adoption of a holistic care approach by nursing staff, helps reduce verbal or physical abuse by caregivers, and minimizes late-stage complications to the greatest extent possible, thereby demonstrating significant clinical utility.

## Data Availability

The raw data supporting the conclusions of this article will be made available by the authors, without undue reservation.

## References

[ref1] Aarsland D. (2020). Epidemiology and pathophysiology of dementia-related psychosis. J. Clin. Psychiatry 81:AD19038BR1C. doi: 10.4088/JCP.AD19038BR1C, PMID: 32936544

[ref2] Ahmed I. Ishtiaq S. (2021). Reliability and validity: importance in medical research. J. Pak. Med. Assoc. 71, 2401–2406. doi: 10.47391/JPMA.06-861, PMID: 34974579

[ref3] Albert M. Cohen C. (1992). The test for severe impairment: an instrument for the assessment of patients with severe cognitive dysfunction. J. Am. Geriatr. Soc. 40, 449–453. doi: 10.1111/j.1532-5415.1992.tb02009.x, PMID: 1634695

[ref4] Besmens I. S. Watson J. A. Akyildiz E. Mundy L. R. Giovanoli P. Calcagni M.. (2025). Comprehensive validation of the German version of the LIMB-Q. Ann. Plast. Surg. 94, 365–369. doi: 10.1097/SAP.0000000000004175, PMID: 39652865 PMC11902580

[ref5] Brislin R. W. (1976). Comparative research methodology: cross-cultural studies. Int. J. Psychol. 11, 215–229. doi: 10.1080/00207597608247359

[ref6] Browning W. R. Yildiz M. Hernandez Chilatra J. A. Yefimova M. Maxwell C. D. Sullivan T. P.. (2024). Mechanisms underlying the use of abusive and neglectful behaviors in dementia caregiving: the role of caregiver mental health. Res. Gerontol. Nurs. 17, 227–236. doi: 10.3928/19404921-20240808-01, PMID: 39347758 PMC13436597

[ref7] Dooley S. Hopper T. Doyle R. Gilheaney O. Walshe M. (2025). Profiling communication ability in dementia: validation of a new cognitive-communication assessment tool. Int. J. Lang. Commun. Disord. 60:e13153. doi: 10.1111/1460-6984.13153, PMID: 39736087 PMC11684355

[ref8] Giannouli V. (2017). Alzheimer’s disease: psychosocial dimensions of a modern plague. Encephalos 54, 33–38. Available online at: https://www.encephalos.gr/pdf/54-2-03e.pdf

[ref9] Giannouli V. (2022). “Elder abuse and victims with disabilities” in Victimology. ed. Gopalan R. T. (Cham: Springer).

[ref10] Giannouli V. (2024). Can changes in financial performance be used in the diagnosis of neurocognitive disorders? A systematic review of findings from Greece. Brain Sci. 14:1113. doi: 10.3390/brainsci14111113, PMID: 39595876 PMC11591921

[ref11] Giannouli V. Tsolaki M. (2022). Elder financial abuse and the COVID-19 pandemic: a call to action through training programmes? Psychiatriki 33, 333–334. doi: 10.22365/jpsych.2022.090 36041403

[ref12] Giannouli V. Tsolaki M. (2023a). Brain volumes and metacognitive deficits in knowledge of self, task and strategies in mathematics: a preliminary pilot one-year longitudinal study in amci patients compared to healthy controls. Diagnostics 13:680. doi: 10.3390/diagnostics13040680, PMID: 36832169 PMC9955851

[ref13] Giannouli V. Tsolaki M. (2023b). What do arithmetic errors in the financial context reveal? A preliminary study of individuals with neurocognitive disorders. Neurol. Int. 15, 743–749. doi: 10.3390/neurolint15020046, PMID: 37368330 PMC10304080

[ref14] Gondim G. M. C. Bedê J. M. S. Martins C. A. da Silva F. V. Silveira B. L. R. Ribeiro V. F.. (2025). Reliability, internal consistency, and validity of the World Health Organization disability assessment schedule (WHODAS) 2.0 among adults with heart failure. Heart Lung 70, 30–35. doi: 10.1016/j.hrtlng.2024.11.003, PMID: 39550897

[ref15] Harrell L. E. Marson D. Chatterjee A. Parrish J. A. (2000). The severe Mini-mental state examination: a new neuropsychologic instrument for the bedside assessment of severely impaired patients with Alzheimer disease. Alzheimer Dis. Assoc. Disord. 14, 168–175. doi: 10.1097/00002093-200007000-00008, PMID: 10994658

[ref16] He L. Ayub A. F. B. M. Amri S. B. (2024). Development and validation of a questionnaire to assess the implementation of physical education programs in Chinese junior high schools. BMC Public Health 24:2387. doi: 10.1186/s12889-024-19844-5, PMID: 39223514 PMC11370090

[ref17] Jean K. R. Dotson V. M. (2024). Dementia: common syndromes and modifiable risk and protective factors. Neurol. Clin. 42, 793–807. doi: 10.1016/j.ncl.2024.05.005 39343475

[ref18] Lacobucci D. Ruvio A. Román S. Moon S. Herr P. M. (2022). How many factors in factor analysis? New insights about parallel analysis with confidence intervals. J. Bus. Res. 139, 1026–1043. doi: 10.1016/j.jbusres.2021.09.015

[ref19] Long Z. Huang L. (2025). Development and reliability and validity test of the sleep health literacy scale for college students. BMC Public Health 25:1257. doi: 10.1186/s12889-025-22455-3, PMID: 40181372 PMC11969716

[ref20] Makri E. Giannouli V. (2022). Cross-cultural cognitive and affective differences in aging: can culture shape the expression and perception of psychopathology in old age? Encephalos 59, 34–43. Available online at: https://encephalos.gr/pdf/59-4-01e.pdf

[ref21] Moermans V. R. Mengelers A. M. Bleijlevens M. H. Verbeek H. de Casterle B. D. Milisen K.. (2022). Caregiver decision-making concerning involuntary treatment in dementia care at home. Nurs. Ethics 29, 330–343. doi: 10.1177/09697330211041742, PMID: 34872409 PMC8958636

[ref22] Myers N. D. Ahn S. Jin Y. (2011). Sample size and power estimates for a confirmatory factor analytic model in exercise and sport: a Monte Carlo approach. Res. Q. Exerc. Sport 82, 412–423. doi: 10.1080/02701367.2011.10599773, PMID: 21957699

[ref23] Niznik J. D. Zhao X. He M. Aspinall S. L. Hanlon J. T. Nace D.. (2019). Factors associated with deprescribing acetylcholinesterase inhibitors in older nursing home residents with severe dementia. J. Am. Geriatr. Soc. 67, 1871–1879. doi: 10.1111/jgs.15985, PMID: 31162642 PMC7456032

[ref24] Noguchi T. Shang E. Hayashi T. (2025). Stigma beliefs and attitudes against dementia and help-seeking intentions in hypothetical early signs of dementia: an observational cross-sectional study of middle-aged and older adults in Japan. Int. J. Geriatr. Psychiatry 40, e70141–e70110. doi: 10.1002/gps.70141, PMID: 40790834

[ref25] Olmsted J. (2024). Research reliability and validity: why do they matter? J. Dent. Hyg. 98, 53–57. Available online at: https://jdh.adha.org/content/98/6/53.full 39658068

[ref26] Peavy G. M. Salmon D. P. Rice V. A. Galasko D. Samuel W. Taylor K. I.. (1996). Neuropsychological assessment of severely demeted elderly: the severe cognitive impairment profile. Arch. Neurol. 53, 367–372. doi: 10.1001/archneur.1996.00550040107020, PMID: 8929160

[ref27] Polit D. F. Beck C. T. (2006). The content validity index: are you sure you know what's being reported? Critique and recommendations. Res. Nurs. Health 29, 489–497. doi: 10.1002/nur.20147, PMID: 16977646

[ref28] Saxton J. (1990). Assessment of the severely impairment patient: descriptionand validation of a new neuropsychological test battery. Psychol. Assess. 2, 298–303. doi: 10.1037/1040-3590.2.3.298

[ref29] Saxton J. Kastango K. B. Hugonot-Diener L.. (2005). Development of a short form of the severe impairment battery. Am. J. Geriatr. Psychiatry 13, 999–1005. doi: 10.1176/appi.ajgp.13.11.999 16286444

[ref30] Sheikh A. B. Javed N. Ijaz Z. Leyba K. Barrett E. Dunn A. (2022). Easing dementia caregiver burden, addressing interpersonal violence. J. Fam. Pract. 71, E1–E8. doi: 10.12788/jfp.0349, PMID: 35259332

[ref31] Shi J. Liu S. X. Li J. W.. (2022). Study on the reliability and validityof the Chinese criteria of health scale for the elderly people. Chin. J. Prev. Med 56, 1809–1814. doi: 10.3760/cma.j.cn112150-20220223-00170 36536570

[ref32] Steinsheim G. Saga S. Olsen B. Broen H. K. Malmedal W. (2022). Abusive episodes among home-dwelling persons with dementia and their informal caregivers: a cross-sectional Norwegian study. BMC Geriatr. 22:852. doi: 10.1186/s12877-022-03569-4, PMID: 36371161 PMC9655791

[ref33] Sun J. Li L. Tian X. Fu Y. (2025). Reliability and validity validation of the Chinese version of the family vitiligo impact scale. BMC Public Health 25:258. doi: 10.1186/s12889-025-21439-7, PMID: 39838342 PMC11752934

[ref34] Tsoucalas G. Bourelia S. Kalogirou V. Giatsiou S. Mavrogiannaki E. Gatos G.. (2015). End-stage dementia spark of life: reliability and validity of the "GATOS" questionnaire. Curr. Alzheimer Res. 12, 179–188. doi: 10.2174/1567205012666150204122635, PMID: 25654507

[ref35] World Alzheimer Report. (2024). Global changes in attitudes to dementia [EB/OL]. Available online at: https://www.alzint.org/resource/world-alzheimer-report-2024/ (Accessed September 20, 2024).

[ref36] Yang Y. Wang J. Tong M. Cheng R. Pan J. (2022). The localization and improvement of the functional status scale and the reliability and validity in very low birth weight infants. J*. Zhejiang Univ. Med. Sci* 51, 603–612. doi: 10.3724/zdxbyxb-2022-0336, PMID: 36581578 PMC10494238

[ref37] Yeom H. E. Lee J. (2024). Validity and reliability of the basic psychological need satisfaction and frustration scale among cancer survivors in Korean healthcare contexts. Healthcare 12:2535. doi: 10.3390/healthcare12242535, PMID: 39765962 PMC11675302

[ref38] Zhang J. Tian M. Xu L. (2024). Reliability and validity of the Chinese version of the contraceptive knowledge assessment scale in college students. BMC Public Health 24:2810. doi: 10.1186/s12889-024-20337-8, PMID: 39402504 PMC11472492

[ref39] Zhao W. Peng L. Zhang X.. (2024). Development and reliability and validity testing of a health comprehensive evaluation scale for elder population in medical institutions of Beijing. J. Cap. Med. Univ 45, 1088–1094. doi: 10.3969/j.issn.1006-7795.2024.06.019

